# Biomarkers on the Icy Jovian Moons: Can Europa Also Provide Insights into Life’s Origin?

**DOI:** 10.3390/life16030489

**Published:** 2026-03-17

**Authors:** Julian Chela-Flores, Doron Lancet, Roy Yaniv

**Affiliations:** 1 The Abdus Salam International Centre for Theoretical Physics, 34151 Trieste, Italy; 2 Fundación Instituto de Estudios Avanzados (IDEA), Caracas 1080, Venezuela; 3 Department of Molecular Genetics, Weizmann Institute of Science, Rehovot 7610001, Israel

**Keywords:** index of biogenicity, missions to Europa, Europa clipper, MASPEX, SUDA, biosignatures, early evolution of life, first biomolecules, GARD model, lipid world hypothesis

## Abstract

Within the payloads of JUICE and Europa Clipper, there are instruments suitable for the search of specific biosignatures that can diagnose life tracks in two ways. The payloads include mass spectrometers capable of measuring isotopic abundances for identifying life, and chromatography instruments testing whether ocean worlds harbor amphiphile mixtures, which would lead to a lipid-first origin of life. In this paper we describe how the two missions may begin to test whether there may be large detectable excursions of stable isotopes of chemical elements on the icy surfaces of the Jovian icy moons that are substantially shifted from their expected isotopic distributions. The detection of an unambiguous signal would suggest a biogenic origin, provided care is taken to exclude abiotic thermal isotopic fractionation. Our suggested tests should be confirmed independently with other techniques. Stable isotope geochemistry on the icy Jovian moons has not yet been thoroughly discussed in the literature. In addition, we enquire whether insights into life’s origin could be retrieved from Europa’s ocean and surface, including the question of the first steps in the evolution of life. Special emphasis has been put on an approach to seek on the surface of ocean worlds chemical phenomena that are rather primitive, such as reproducing lipid micelles as roots of protocells, but nevertheless can predict a path towards life with published models.

## 1. Introduction

It is obvious that life begins with chemistry. A central aim of most missions is to discover signatures of present life at the research’s astronomic targets. It is hard to decide when chemistry becomes life, so it is important to carry out scientific studies. We discuss the Icy Jovian moons, especially Europa, as fertile ground for the search of biosignatures. We consider whether upcoming discoveries suggest that life could emerge, answering the question of which were the first biomolecules that led to life as we know it. The Russian biochemist Aleksandr Oparin first published his The Origin of Life in 1924, translated to English in 1938 [1]. In that book, Oparin dealt with the problem of life’s origin, for the first time, from a materialistic perspective. His theory puts forward the production of organic molecules on the early Earth followed by chemical reactions that produced increased organic complexity. His central idea is the spontaneous formation of coacervates—unique droplets containing different organic molecules, including certain organics, polymers and lipids, hence allowing the formation of mutually interacting networks and eventually undergoing mutation and selection. The spirit of this paper is strongly based on Oparin’s teachings.

Many issues of the structure and formation of the Earth include the early formation of its stratification, and the origin and evolution of life cannot be referred to merely in the outline of the analysis of terrestrial matter. To solve these problems, it is important to get the composition and structure of the material in the solar system, e.g., planets and meteorites. A central method for deciding whether specific organics were in the past within a life organism is scrutinizing the isotope ratio values in various samples [2]. To study the isotope fractionation of various elements, beta factor calculations can be effectively applied [3]. Finally, a knowledge of beta factor mass-fractionation is a crucial step in assessing isotopic disequilibrium, a prevalent occurrence that is progressively used to elucidate temporal relationships in geological systems [4].

## 2. Probing for Chemical Elements and Their Isotopes on the Icy Surfaces of Jovian Moons

Potential biosignatures, for instance, the recent report concerning a sample collected by NASA’s Perseverance Mars rover from an ancient dry riverbed in Jezero Crater, could preserve evidence of ancient microbial life. However, like the biosignature proposal in this work, additional studies are required before such biosignatures could be accepted to be related to living organisms [5]. Extending the isotopic fractionation from the rock record to astrobiology has been attempted by means of δ^34^S, which is used in the geologic record [6].

We found it profitable to use isotopic fractionation excursions in astrobiology but attention in previous research projects has been restricted exclusively to the icy surface of the ocean worlds (OWs), which are celestial bodies (planets or satellites) that contain a hydrosphere with abundant water in the form of oceans, often submerging dry land. In the case that concerns us in this review, the Jovian and other outer solar system moons, OWs are also called icy worlds, which is when the land has been totally submerged. This is unlike our own planet, where continents are present.

We have not entered into the wider context of isotopic fractionation taking place in planetary science, since that would clearly require a more extensive discussion. For example, the Life Detection Knowledge Base (LDKB) has been developed by a large group of researchers in order to test and evaluate strategies to search for evidence of life beyond Earth. Their emphasis has been on recognizing potential false-positive and false-negative results in the general case of planetology, rather than on the more restricted case of the icy surfaces of ocean worlds which is exclusively the main topic to which this review has been dedicated [7,8]. The LDKB authors have done careful analysis about applying life detection criteria to assess isotope abundance patterns as potential biosignatures. They have used previous work to create a database to put some order into planning future missions. With LDKB it will be easier to catalog reliable biosignatures from the many that are likely to be proposed after JUICE and Europa Clipper begin their measurements. At the same time, they have provided a repository for the acquisition of new data. These authors have introduced useful language for highlighting reliable biosignatures, “high and low arguments”. For instance, isotopic discrimination during biological dissimilatory sulfate reduction can yield delta S values of sulfides that are 15–72 (‰), which is lower than the reference values δ^34^S (‰) of their sulfate source. In Section 6 below we shall return to the clear example of such isotopic fractionation due to Wortmann and coworkers.

For the ongoing missions to the Jovian system [9,10], sulfur would have the most relevant excursion of isotopic fractionation for the detection of traces of biogenic activity compared to other biogenic elements: carbon, hydrogen, oxygen, nitrogen and phosphorous [11]. Sulfur consists of four stable isotopes: ^32^S (95.02%), ^33^S (0.75%), ^34^S (4.21%) and ^36^S (0.02%). Bacterial sulfate reduction (BSR) is a well-understood process, in which the involved bacterium links atoms of hydrogen with sulfur taken away from the dissolved sulfate (SO_4_^2−^) of seawater to form hydrogen sulfide (H_2_S). This gas is enriched in ^32^S relative to the seawater source [12].

We consider the redistribution of the primordial isotopic mixtures as usual in terms of the following sulfur delta parameter [13]:

δ^34^S = [(^34^S/^32^S)_sa_/(^34^S/^32^S)_st_ − 1] × 10^3^ [‰, VCDT] (1)

The value of δ^34^S is taken to be zero when the sample coincides with the corresponding value of the Canyon Diablo meteorite (VCDT), a standard troilite (FeS) that was found in Phoenix, Arizona. The useful parameter δ^34^S allows a comparison of a sample (sa) with the standard (st) VCDT. The relevant terms are the dominant sulfur isotope (^32^S) and the next in abundance (^34^S). In fact, the quotient (^34^S/^32^S)_st_ is the average terrestrial fraction of the two most abundant isotopes of sulfur. In a given sample, we obtain a positive value of δ^34^S when the quotient (^34^S/^32^S)_sa_ exceeds the value of the standard VCDT; otherwise, we obtain a negative value when the quotient (^34^S/^32^S)_sa_ is less than the value of the standard VCDT.

Analogously, the biogenic element chlorine has the following two stable isotopes [14]:

^35^Cl (75.78%) and ^37^Cl (24.22%)

The Cl isotope fractionation is discussed in terms of δ^37^Cl, which is defined in the same way as above for the δ^34^S parameter of sulfur. Measurements of the relative abundance of Cl isotopes is reported in the per mil notation (‰) vs. Standard Mean Ocean Chloride (SMOC). SMOC is thought to be isotopically homogeneous to within ±0.15‰ [15]. The justification of extrapolating the relative abundance of Cl to Europa is suggested by the work of S. Trumbo and his co-workers [16], after the earlier work of others [17].

Interestingly, within our overall objective of searching for reliable indices of biogenicity, the bacterium *Azospira suillum* shows a significant stable isotope fractionation excursion of −15‰ [18,19]. Chlorine has been identified on the icy surface of Europa [16,17]. The model assumes that Europa’s seafloor was initially chondritic Type II. Consequently, the water–rock cycling at the silicate seafloor is capable of producing a chloride-rich ocean [20,21,22].

With the sulfur isotopes, it is possible to report three measurable delta values: δ^33^S, δ^34^S and δ^36^S. The delta parameter, δ^34^S, has been highlighted for a very long time in the terrestrial rock record as a valuable index of biogenicity, but only more recently in the context of astrobiological discussions, as in our previous papers reported in this review. The support for δ^34^S distributions as indices of biogenicity has been extensively illustrated from sediments throughout the terrestrial rock record. The extrapolation from geophysics on Earth (δ^34^S distributions) to the Jovian moon Europa has its rationale to a large extent based on Manfred Schidlowski and his geochemical co-workers [6]. Examples range throughout the whole evolution of life on Earth, for instance:

1. Pyrites in banded iron formations (BIFs) from the Isua Greenstone Belt dating from the Archaean in the time interval of 3.7–3.8 Gyr BP, which have been assigned a δ^34^S (‰) of −4 ± 2 [23].
2. A second example is from a rock formation in the Pilbara region of Western Australia, in which barite deposits of some 3.47 Gyr BP have been assigned values of δ^34^S of −5 ± 7 [24].
3. A third example is given by pyrites in black shales from 2.7 to 2.8 Gyr BP from Western Australia, which have a δ^34^S of −8 ± 7 [25].

Finally, pyrites and other sulfides from the beginning of the Triassic Period from Central Europe were characterized by a δ^34^S of −40 ± 20 [26]. We should underline that the above examples range from the earliest manifestations of life in the Archaean to a significantly recent geologic time, namely, the Triassic Period 251.9 Myr BP, when multicellular life was widespread.

In fact, both chemical elements, sulfur and chlorine, on the surface of the Jovian moon Europa and whose origin is not of an external source could have been cycled by oceanic dwelling microorganisms. This hypothesis will be available for testing after the arrival of the above-mentioned NASA and ESA missions, provided that the sulfur fractionation due to metabolic modifications that may have taken place underneath the icy shell render such phenomenon measurable. Fractionated sulfur isotopes may reach the upper side of Europa’s icy shell, since for some time we have been aware with telescopic observations of the existence of water vents arising from the Europan ocean. In other words, if microorganisms were responsible for sulfur fractionations in the Jovian OWs, their activity could be detectable from icy shell surficial traces with the sensitivity of the available instrumentation that has been incorporated in the payloads of JUICE and Clipper. To follow this approach for the identification of biosignatures on the icy surface of Europa and other OWs, some instrumental and technical challenges remain. These will be briefly outlined in Section 4 below.

## 3. The Identification of Fractionation Due to Abiotic Processes

Thermochemical processes are the first topic to keep in mind regarding the Jovian icy moons from the point of view of stable isotope geochemistry. Recent attempts have been restricted to anticipating how instrumentation could be used for the search for habitable ecosystems in the exploration of Europa and Ganymede [27]. Additionally, previous discussions on how living processes can be distinguished from abiotic processes were kept in mind [28]. Such discussions followed the standard approach, as briefly described in this section.

In the fractionation of stable isotopes, there are two mutually exclusive regimes: sulfate reduction in the range 60–80 °C should be compared with high temperatures in the range of 150–200 °C. These higher temperatures are generally larger than the cases of biogenicity. However, the above two thermal regimes do overlap when aqueous sulfate can be reduced by organic compounds at temperatures close to the water boiling point [29]. It seems reasonable to claim that abiotic fractionations are largely irrelevant for Europa, since abiotic sulfate reductions are larger than biogenic ones for Europan ocean considerations [30].

More specifically, experiments have only yielded fractionations of 10–20‰ for temperatures in the range of 100–200 °C. There is some ocean–seafloor contact, hence raising the possibility of abiotic fractionation due to the exposure to the temperatures of hydrothermal vents. However, we should keep in mind that at the seafloor the emergence of microorganisms would be subject to thermochemical sulfate reduction, but globally this would be largely negligible from the experimental evidence of fractionations as it would be smaller than about 20‰ [31].

When a chemical element is reduced due to heat rather than by microorganisms, we should insist in the use of the abbreviated phrase “thermochemical sulfate reduction (TSR)”. This adoption allows us to distinguish this abiotic phenomenon from the following independent sources of abiotic fractionation. Next in importance are the hydrothermal processes. The capital delta parameters ^33^Δ and ^36^Δ are defined as follows [32]:

^33^Δ = δ^33^S − [(^34^R_SAMPLE/_^32^R_VCDT_)^0.515^ − 1] (2)

^36^Δ = δ^36^S − [(^34^R_SAMPLE/_^32^R_VCDT_)^1.90^ − 1] (3)

where ^3x^R = ^3x^S/^32^S relative to the standard VCDT [Vienna Canyon Diablo Troilite (FeS)].

The capital delta parameters measure deviations from mass-dependent relationships [33], which are compared with biogenic sulfides. It should also be remembered that there are small contributions, or even null contributions, from microbial sulfate reductions into hydrothermal sulfides at sediment-free mid-oceanic ridge systems. Measurements of δ^x^ parameter (1), where x = 32, 33, 34, and 36, were carried out in four high temperature seafloor hydrothermal vents along sediment-free ridge systems. The sulfur systematics suggest isotopic exchange between sulfate and sulfide at T = 400 °C. The systematics can decouple microbial sulfur cycling at ocean crust–seawater interfaces [34].

Photochemical processes should also be taken into account. Returning to the above two thermal regimes, there is some overlap between them, but these cases are mostly irrelevant for our main interest that concerns Europa. We should also keep in mind that at the seafloor the possible origin of life would be subject to TSR, since such fractionations have been verified to be smaller than about −20‰ [35].

Careful experimental surveys of SO_2_ photolysis caused by a series of different UV wavelengths have uncovered a relationship between such wavelengths and sulfur isotope fractionation in the geological record, as in the above work of Farquhar and co-workers. These experiments have demonstrated that there is a given wavelength for which SO_2_ photolysis coincides with certain Archaean samples: other wavelengths in these experiments differ from the observed geological record. This can be used to retrieve information from the terrestrial atmosphere. To sum up, there is a certain wavelength-sensitive, mass-independent abiotic sulfur isotope fractionation effect during SO_2_ photolysis.

It is interesting to highlight the fact that biotic processes that are characteristic of hydrothermal environments can simulate the isotopic discrimination exhibited by sulfate-reducing enzymes. For instance, in hydrothermal systems, differences in δ^34^S values between sulfate and sulfide of −14‰ to −21‰ have been shown to occur via geochemical (abiotic) processes [36]. These values are too small to be relevant, for if observed, the magnitude of the δ^34^S necessary to be significant (δ^34^S > −60 to −80‰) would in this case be suggestive of a biogenic origin. Oceanic crust sulfur-cycling constraints have been investigated by Jakob Ciazela and co-workers [37]. These investigations allow us to grasp more intimately the source of abiotic sulfur presence in the ocean.

Indeed, research on geological processes at slow-spreading ridges specifically details how melt inclusions and gabbroic veins are affected by the assimilation of hydrothermally altered lithosphere enriched with volatiles. Extrapolating these studies to the possible events that take place at the bottom of the Europa ocean, they suggest that abiotic sulfate can be introduced into the Europa ocean. But we should not overlook that abiotic isotope fractionation is known to take place at high temperatures, who normally do not occur on Europa except at the ocean floor.

Exceptionally, care is required in the biogenic interpretation of some surficial sulfate (whether in the leading or trailing hemispheres). Such fractionated sulfur may be of abiotic origin if isotopic sulfate fractionation is detected on the surface of Europa that had originated originating from its sea floor.

## 4. Fractionation from the Rock Record to the Jovian Moons

Our initial suggestion was to point out the relevance of transferring to the astrobiological context the successful application of the geochemistry of stable sulfur isotope fractionation from the early rock record on Earth. Our motivation for this work is to address several questions, the main ones being [38]: Sulfur isotopes are the main objective of the search for biosignatures. In addition, it would be better to have a combination of sulfur and carbon isotope anomalies.

Sulfur is unique amongst the biogenic elements, since in extraterrestrial material (lunar fines and meteorites) [13] sulfur rather than carbon shows in isotopic fractionation a narrow range of values of about zero per mil. An additional question in favor of sulfur isotopic fractionation is to accept some contribution of sulfur from Io, and still find the biogenic fraction in those sulfur deposits.

Since we are assuming from the data that only biogenic processes alter the null values of the isotopic sulfur fractionation, the contribution from the exogenous (Io) sulfur, if it were the product of bacterial metabolism, would not give the significant biogenic signal that would otherwise be produced by endogenous sulfur [38]. Testing for the detection of S isotope biosignatures on Europa is a relevant topic. There are two sources of surficial sulfur, not only exogenous S coming from Io, as we have just mentioned, but models also suggest that sulfate can have its source from the ocean [39,40,41]. In any case, due to the instrumentation on the Europa Clipper and JUICE missions (cf., Table 1) we will have the possibility to identify key syntheses on sulfur isotope biosignatures. Although abiotic fractionation occurs at relatively high temperatures that are absent on Europa except at the bottom of its deep ocean, arguing in favor of surficial sulfate from the leading and trailing hemispheres is necessary either from models or observations. Moreras-Marti and colleagues provide a list of key syntheses on sulfur isotope biosignatures [32].

We have attempted to go from fractionation in the rock record to Galilean moons. Besides long-term planning for the exploration of the moons of the Jovian system, additional searches for biosignatures should focus firstly on two of the Galilean moons: Europa and Ganymede. Subsequently, Callisto would also deserve a separate mission too. Fortunately, both missions by ESA and NASA that are already on the way to the Jovian System are equipped with appropriate instrumentation [9,10]. In the short term, such instrumentation makes it feasible to explore the exoatmospheres of the Jovian OWs. Since generally Europa’s surface composition determines the composition of its atmosphere and can yield information on surface components, most surficial trace species will be present in the extended atmosphere [42].

In view of the many instruments that are in the payloads of the forthcoming missions to the Jovian system with the δ^34^S index of biogenicity, it is convenient to further comment on only a few of them that would be relevant from the point of view of the biogeochemical biosignatures. Firstly, there are mass spectrometers: in JUICE the PEP Package with its NIM instrument (University of Bern, Bern, Switzerland); secondly, in the Europa Clipper we have MASPEX (Southwest Research Institute (SwRI), San Antonio, TX, USA) and SUDA (University of Colorado Boulder, Boulder, CO, USA). The NIM instrument is intended to measure the exospheres of the Jupiter satellites during their flybys [43]. For simplicity we have added Table 1.

**Table 1:** Relevant for habitability issues of NASA Europa Clipper and ESA.

	MASPEX [44]	NIM [45]	SUDA [46]
Agency	NASA	ESA	NASA
Resolution	achieving over 25,000 M/ΔM	M/ΔM > 1100	Roughly 200–250 M/ΔM
Mass range	1–1000 u	1 to 1000 amu (atomic mass units).	1–250 atomic mass units
Isotopic capability	Sensitivity for detecting trace volatile isotopes and complex organics, with a dynamic range >5000	expect an accuracy of 1% for the isotope measurements	The precision of the isotopic ratios in individual mass spectra will generally be quite low

**MASPEX:** Sulfur isotopes have a relatively large negative mass defect which means that the fragment ions containing sulfur are easily separated from most CHON ions as these have a positive mass defect. A mass resolution of ~5000 should be sufficient to unambiguously measure S-isotope fragment ions [44]. The remaining variables will be their abundances—they must be above the detection limit—and the relative abundances of nearby fragment ions. Significantly higher abundances of nearby fragment ions can be dealt with to a limited degree by increasing the resolution, but then there is also the possibility of coalescence for example. In other words, MASPEX has the capability but the Europa composition must comply. Clearly, this statement applies to our ability to measure any given molecules/elements. The answer to measuring S-isotope fractionation is primarily dependent on the quality of the data that will be obtained for making precise measurements of the different isotopes. So again, it comes back to the abundance of S-bearing species, their isotopes, and the relative abundances of interfering ions.

**NIM:** Regarding the achievable isotopic precision, given sufficient signal we expect an accuracy of 1% for the isotope measurements. Better accuracies can only be achieved by having reference gases available in the instrument, which is not the case for NIM. For example, going down to a 50 km closest approach instead of the 400 km current baseline would make sulfur isotopes available (via SO_2_ and perhaps H_2_S) [45].

Due to the required signal-to-noise ratio, the smaller isotope has to be measured with an S/N of 100.

With respect to plausible sampling matrices (sputtered exosphere, plume grains, surface ice), including contamination by radiolysis products, the following must be considered. For the thermal exosphere there will only be the H and O isotopes for which an accurate ratio can be measured. That is limited by the flyby distances of JUICE at closest approach. For the plumes, many more species should be available for isotope measurements, depending on the chemical composition of the plume. Signals from the plume will be orders of magnitude higher if it is possible to fly through a plume.

Ice grains would give a good signal as well when they evaporate in the ion source, but the probability that an ice grain will enter NIM is low given that it is built for measuring the exospheric gas. For comparison, with instruments on Rosetta, nine ice grains were measured during the about 2-year period close to the comet.

**SUDA**: In principle, we can derive the ion counts of the sulfur-bearing ice grains as they make impact [46]. The difficult part is creating sulfur-bearing ice. Currently, ice layers are created for impact experiments by depositing vapor on cryogenic targets. Simply adding SO_2_ to water, for example, will not produce a mixture of sulfur and water ice because of phase separation in the vapor. Conversely, creating pure SO_2_ ice from SO_2_ gas raises safety concerns due to the risk of inhaling SO_2_, which forms sulfuric acid in the lungs.

There is another aspect to consider. SUDA records mass spectra of individual ice grains whose sizes range from a few tens of nanometers to a few microns—the sample sizes are tiny. Typically, the abundance of minor species in the ice matrix varies from grain to grain. The precision of the isotopic ratios in individual mass spectra will generally be quite low. However, tens of thousands of mass spectra during a flyby can be collected. Assuming that all the spectra provide information on the same isotopic abundance of sulfur, the precision derived from the combined spectra can be quite high.

In the present context we could raise the question of whether the bioastronomy of OW biosignatures is relevant, for we still do not have a reliable SETI signal. Let us now consider some of its consequences. As a working hypothesis, we could assume that the evolution of life in the universe can be explained in terms of evolutionary forces that we experience today. In that case, once the living process has started, it will continue due to evolutionary pressures, as it was explained in detail at the Hawaii Bioastronomy conference [47]. However, there is little doubt that life’s origin, at its roots, derives from known physical and chemical laws of nature. The most relevant scientific framework is the laws of thermodynamics, in which life is a set of out-of-equilibrium phenomena, involving thermodynamic dissipation [48,49].

We can devise experiments addressing specifically the search for the evolution of microorganisms, especially from prokaryotes to eukaryotes, with the latter being a first step towards the evolution of intelligence as we have witnessed on Earth. Indeed, several arguments militate in favor of the ubiquity of eukaryogenesis in habitable environments in other worlds, as argued at the San Juan Capistrano Conference No. 5 [50]. We had argued that in the Drake Equation a factor f_e_ should be included, which denotes the fraction of planets or satellites where eukaryogenesis occurs [51].

For the distribution of life in the universe, one approach is the search for microorganisms to test for the onset of eukaryogenesis in the solar system. Alternatively, the direct approach is a major aim of bioastronomy. Specifically, the time-honored SETI search for (intelligent) life in other solar systems, as proposed by Frank Drake in 1960 when he set out to search for extraterrestrial intelligence as a legitimate and doable scientific endeavor [52], which was subsequently widely accepted by the bioastronomy community in terms of the Drake Equation. Sadly, as underlined at the beginning of this Section, this approach has still not produced a reliable signal, suggesting that alternatives should be followed up, such as searching for OW biosignatures as indicated in this review.

## 5. The Future of Jovian Ocean World Exploration

In an astrobiological context of biosignatures from the Europan ocean, the minor S isotopic biosignatures are not corrections of an order of magnitude comparable with the biosignatures in the terrestrial cases, where there are definite large fractionation excursions of up to 72‰ that have been caused by in situ microbial sulfate reduction [53]. Provided the results of the orbital Jovian measurements gave sufficiently large fractionation excursions (>50–60‰, or larger), the measurements of large δ^34^S could be interpreted as a biosignature. We have insisted that measurements of the Jovian orbital missions ought to search for the presence of significant large values of δ^34^S (numerically, of the order of 70‰ or larger; this is justified because such large fractionation excursions can experimentally be clearly distinguishable from a null result that would mean no fractionation excursions).

With the two ongoing probes for Europa or Ganymede, or with a subsequent lander on any of the OWs of the Jovian system, the isotopic abundances discussed in the present review should be complementary with other searches for life. Eventually, the measured anomalous isotopic abundances could be a first step towards an early identification of the existence of other life away from planet earth, possibly when JUICE and Clipper begin gathering data in the 2030s, or later.

An eventual Europa lander remains an appealing project for NASA [54]. This is not a new concept, since early proposals were made at the end of the twentieth century, in which a probe called a cryobot capable of penetration through the icy shell of the Jovian moon and provided with a miniature submarine probe (hydrobot) could attempt to explore the Europan ocean.

Such a proposal initiated at JPL was appealing to some of us at that time [55]; but at present, it is not clear whether a Europa lander would be capable of carrying a hydrobot in its payload and deliver it into the ocean. With our present technology and especially with the current limited financial support, this possibility is definitely to be excluded in the short term, but it should not necessarily be discarded in the long term. Yet more recently some more advanced proposals have been tentatively explored with significant financial support, such as an extension of the cryobot miniature surficial probes such as PRIME which could be the comprehensive cryobot architecture for accessing Europa’s ocean [56].

To conclude with Europa landers, some recent more realistic proposals focus on Europa lander instruments. It is suggested to couple a mass analyzer with a sample introduction/laser ablation/desorption ion source (ORIGIN instrument).

This instrument is more like LMS with the laser ablation/desorption ion source [57]. But the work of Pitesky and Hand summarize a further series of ideas that ought to be followed up after the “post-Europa Clipper” era.

## 6. Instrumentation Challenges, Uncertainties and Implicit Hypotheses

**Instrumentation Challenges:** It is intuitive to specifically consider the instruments on JUICE and Europa clipper, but there remains the challenge of demonstrating how these instruments could determine the sulfur isotopic ratios and if the results would be accurate enough. For example, NIM on JUICE is expected to have only a mass resolution of around 800.

In the long term, it is uncertain whether penetrating instruments (cryobots) may be an option to get further insights of the chemical nature of the ocean to constrain the distribution of chemical elements on the OWs icy surfaces.

**Uncertainties:** A mass resolution of around 800 is not sufficient to differentiate, e.g., between H_2_O_2_, ^18^O^16^O,^34^S, and H_2_S, which all have the nominal mass 34. Radiolysis/photolysis species could be expected in realistic observation scenarios and thus should be considered. The mass resolution of SUDA is even less than that of NIM. Only MASPEX has a sufficient resolution to allow delineation of the species at nominal mass 34. Our suggestion is only to be interpreted as a first step towards potential reliable biosignatures. Our definite conclusion is that although there are some uncertainties, the instrumentation for the payloads of the Europa Clipper mission is generally appropriate to address the question of identifying potential biosignatures.

**Implicit Hypotheses**: Related is the question of how the (heavy isotope-enriched) sulfate anions reach these mass spectrometers. For SUDA, which has the lowest mass resolution, it seems intuitive. It is less obvious for NIM or MASPEX.

Another implicit hypothesis is whether all the sulfur on the icy surfaces of the ocean worlds, including the specific case of Europa, is exogenic. There remains the question of whether the sulfur that could emanate from hydrothermal vents could reach the lower side of the icy shell and eventually contaminate the exogenic sulfur, whose source could be exogenic, possibly emanating from the Jovian moon Io’s significant volcanic eruptions.

Tentatively there has been a report of the detection of a characteristic NH_3_ absorption feature at 2.20 ± 0.02 µm on Europa’s icy surface by careful studies of the NIMS spectrometer that suggests NH_3_-hydrate and NH_4_-chloride are the most likely candidates. The implication is that there has been emplacement from the underground (or shallow subsurface). The paper suggests transportation to the surface via cryovolcanism during the recent geological past [58]. The presence of ammoniated compounds implies a thinner ice shell [59] and a thicker, chemically reduced, high-pH subsurface ocean on Europa [60]. Besides reporting the detection of NH_3_-bearing components, this study also presents the first evidence of a N-bearing species on Europa, an element that is intimately related to life as we know it on Earth, especially for the synthesis of more complex biomolecules such as amino acids, nucleotides and phospholipids.

## 7. Discovering Evidence for Future Life on Ocean Moons

There are in fact two quite different approaches for finding chemical entities on planets or their satellites. The first is the attempt, as in the above Section 2, Section 3, Section 4, Section 5 and Section 6, to discover chemical phenomena suggesting that life is currently existing on the planet or their moons, or that life existed there in the past. The second approach, to be reviewed in the present Section 7, consists of seeking chemical phenomena that are rather primitive but when combined with published models can predict a path towards life. In the previous sections, we discussed evidence for past or present life on icy Jovian moons, especially Europa, as fertile ground for the search of biosignatures. Here, instead, we consider whether upcoming discoveries from the current missions to the Jovian system or later could suggest that life could emerge. This could help to answer questions about the early stages of life’s emergence on Earth.

**Figure 1:**
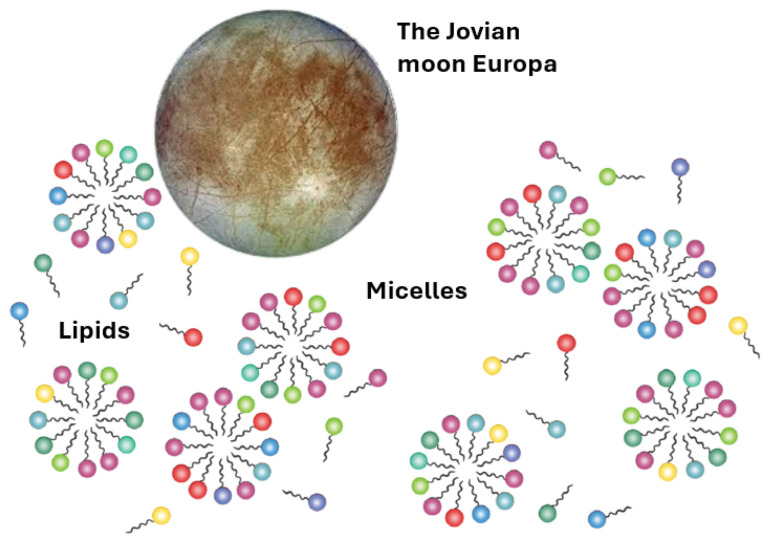
On planet Earth, with a repertoire of 100 lipid types, an assessment computes via the area of oceans, thickness of populated top layer, and size and acceptable micellar concentration [64] a huge library of 10^33^ micelles (nanoscopic protocells), each with a different composition (different colors represent different lipid types) [64]. With a 4-fold smaller diameter of the surface, the micellar library size will be about one order of magnitude (~10^32^). As the GARD model of micellar self-reproduction probability is computed as P = 10^−12^ [65], a yet astronomic number of 10^20^ micelles on Europa might undergo compositional self-reproduction. The instrumentation on future expected Europa missions would be able to detect micelles and measure their composition. This will allow one to find out if in limited environments in the ocean of Europa there are proto-species with numerous micelles having similar composition (with only a few mutations) [66]. Such an observation would possibly indicate a seed of Darwinian evolution at life’s origin on Europa.

In line with the lipid world hypothesis [61], which is realized and quantitated by the GARD model, a group of amphiphile aggregates, e.g., micelles or vesicles, may be protocell precursors [62]. The most important findings portrayed by computer simulations show that some of these aggregates can undergo self-reproduction [63,64] (Figure 1).

**Figure 2:**
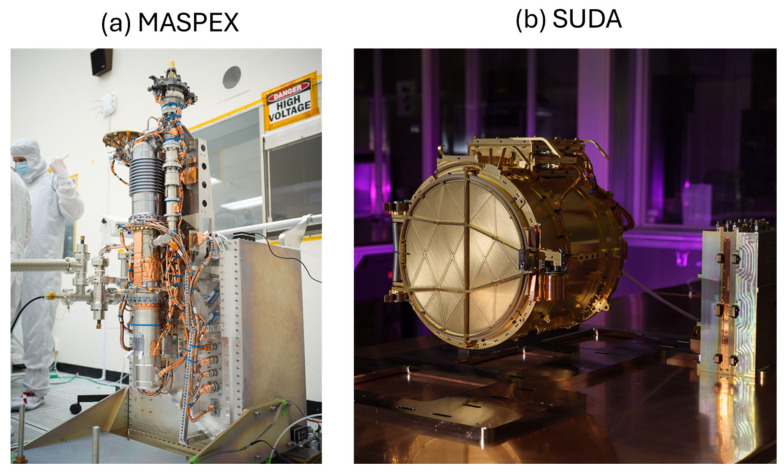
This instrumentation is carried on the Europa Clipper mission. (**a**) MASPEX (Mass Spectrometer for Planetary EXploration) measures volatile and organic molecules, including amphiphilic lipids in Europa’s sputtered exosphere as well as ocean plumes. (**b**) SUDA (SUrface Dust Analyzer) is a dust/ice mass spectrometer, analyzing salts and organics in grains. The results would possibly support the lipid world origin [61] (see Figure 1).

Self-reproducing amphiphile assemblies as described by the GARD model provide compartments that concentrate reactants and sustain coupled catalytic networks. Once such compartmentalized systems incorporate redox-active chemistries [67] then sustained sulfur cycling can emerge, and the associated kinetic isotope effects would generate measurable δ^34^S fractionations as geochemical signatures of evolved protocellular metabolisms. We now intend to discuss how the instrumentation currently included in Europa Clipper’s payload can support the lipid world hypothesis. The Clipper’s Mass Spectrometer for Planetary EXploration (MASPEX) can generally measure volatile and organic molecules in Europa’s sputtered and radiolytically processed exosphere as well as ocean plumes at very high sensitivity and resolution [68]. This is the best shot at detecting amphiphilic organics, beginning with fatty acids, and ending with rather complex lipid-like molecules that can aggregate into micelles and vesicles. The SUrface Dust Analyzer (SUDA), which is a dust/ice grain impact time-of-flight mass spectrometer, analyzes ice grains/ejecta and is particularly strong at salts and organics in grains, which has importance for reporting counterions and salinity levels that set aggregation behavior, i.e., critical micelle concentration (CMC) [69] (Figure 2).

Taken together, both technologies can also help find chain-length distributions and headgroup diversities that can define amphiphile populations rather than random trace organics. The above information allows for determining a whole spectrum relevant to protocellular chemistry. Careful analyses of the data and discovering amphiphiles in non-equilibrium ratios should provide further robustness. All in all, the data collected could provide support for the idea that ocean worlds could harbor amphiphile mixtures or populations that would lead to a lipid-first origin of life. Results from the current mission could also guide future landers, including sample-return missions.

To demonstrate reproduction or heredity or infer specific structures (e.g., micelles or vesicles) directly, there is a need to show high similarity of composition and that they are progeny of a composome (attractor-like reproducing state predicted by the GARD model) reproducing with only a few mutations. Such groups may be considered protospecies, and hence may be viewed as a seed of life capable of undergoing natural selection, the prerequisite for Darwinian evolution.

A central requirement for evaluating compositional heredity in Europa samples in future endeavors is the ability to resolve the molecular composition of lipid micelles after controlled growth–dilution cycles, allowing the detection of composomes. This necessitates instrumentation capable of handling microliter-scale melted-ice aliquots, separating complex amphiphile mixtures, and quantifying their relative abundances with sufficient resolution to distinguish non-random chemical organization from stochastic assembly. These include: space-qualified microfluidic capillary electrophoresis (CE) platforms, most notably the “Chemical Laptop” model, a fully automated CE–LIF (capillary electrophoresis–laser-induced fluorescence) system for high-resolution organic separations [70,71] and the more recent Microfluidic Organic Analyzer (MOA) [72], which integrates a programmable microfluidic handler with a precision CE wafer. The latter is explicitly targeted to ocean-world missions, providing robust separation of amphiphiles by charge and size and converting each micelle into a measurable electrophoretic composition vector. Complementary detection can be achieved using miniature laser-desorption mass spectrometry (LDMS) [73], in which micelles are disrupted and their components resolved by mass-to-charge ratio, yielding quantitative stoichiometric spectra. Front-end sample preparation—including melting, filtration, derivatization, reagent addition, and aliquoting—is supported by monolithic lab-on-chip processors such as SPLIce [74], while auxiliary techniques such as contactless conductivity microfluidics for ionic and amphiphile profiling [75] and digital microfluidics for controlled mixing, splitting, and serial growth–dilution cycles [76] provide additional operational flexibility. Together, these microfluidic and spectrometric technologies form a realistic, flight-deployable analytical architecture. In this, micellar compositions can be reconstructed across multiple aliquots and compared statistically to the compositional clustering expected for GARD-type composomes.

**Figure 3:**
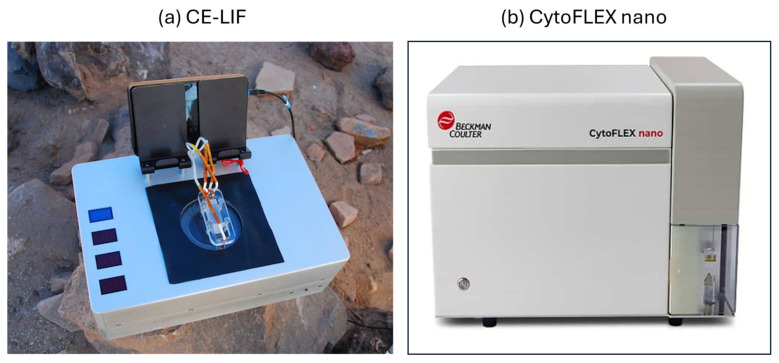
(**a**) A portable instrument using Capillary Electrophoresis Laser-Induced Fluorescence (CE-LIF) technology is being developed and tested by NASA’s Jet Propulsion Laboratory (JPL) (Pasadena, CA, USA) for future missions to search for evidence of extraterrestrial life. (**b**) CytoFLEX is a nano-type flow cytometer that would analyze potential living cells and also nanoscopic reproducing micellar protocells [62,64] (see Figure 1).

In parallel, new technologies are being utilized in terrestrial settings that have, with modification, potential to be employed in extraterrestrial settings as well. One such technology, termed CytoFLEX nano flow cytometer, is currently in use to profile extracellular vesicles [77,78] and lipid nanoparticles (LNPs) [79,80]. While at this point in time the CytoFLEX nano flow cytometer is better suited for >80 nm micelle profiling, in the near future this might be honed down to also suit smaller sizes. Providing it is further fine-tuned to a space-qualified nanoscale cytometer, the advantage of such instrumentation would be direct detection of single-particle biosignatures as opposed to bulk composition detection with CE-LIF. Although the sensitivity for micelles is borderline, the next step in evolution, i.e., the fusion of micelles to much larger vesicular protocell precursors (0.1–10 µm) [63] and that both micelles and vesicles can show the emergence of protospecies, could be significantly supported by the above-mentioned technology (Figure 3).

## 8. Conclusions

We have suggested that the search for biosignatures on Europa’s icy surface should aim at the search for a high argument signal, in the LDKB terminology, by identifying a definite S-isotopic excursion. Such potential biosignatures, if detected, should be supplemented soon by independent evidence to produce complementary evidence of truly a biogenic origin. In other words, the ongoing Jovian orbital missions in due course could search for large values of δ^34^S for the possible detection of meaningful values.

This could occur either orbitally, or eventually by means of a lander. Thus, such results would be a first step for the detection of a possible biosignature, to be confirmed by independent measurements. Some possibilities have been explored by looking into the early stages of life on earth. The astrobiological experimentation described in Section 7 would be useful for discovering chemical entities on oceanic moons, protospecies that could be evidence on the surfaces of Europa and Ganymede for a route to the future emergence of life.

## Data Availability

No new data were created or analyzed in this study.

## Abbreviations

The following abbreviations are used in this manuscript:

BIF	Banded Iron Formation
BP	Before the Present
BSR	Bacterial Sulfate Reduction
CE	Capillary Electrophoresis
CDM	Canyon Diablo Meteorite
CMC	Critical Micelle Concentration
CytoFLEX	Cytometer, Flexible (nano flow cytometer)
ESA	European Space Agency
GARD	Graded Autocatalysis Replication Domain
LIF	Laser-Induced Fluorescence
LDKB	Life Detection Knowledge Base
LDMS	Laser-Desorption Mass Spectrometry
LIMS	Laser Ablation Ionization Mass Spectrometry
LMS	Laser-Ablation Time-of-Flight Mass Spectrometer
LNPs	Lipid Nanoparticles
MASPEX	MAss SPectrometer for Planetary Exploration (Europa Clipper)
MOA	Microfluidic Organic Analyzer
MSR	Microbial Sulfate Reducers
NASA	National Aeronautics Space Administration
NIM	Neutral Ion Mass Spectrometer (JUICE)
NIMS	Near-Infrared Mapping Spectrometer (Galileo)
ORIGIN	ORganics Information Gathering Instrument
OW	Ocean World
PEP	Particle Environment Package (JUICE)
PRIME	Probe using Radioisotopes for Icy Moons Exploration
SETI	Search for Extraterrestrial Intelligence
SIG	Stable Isotope Geochemistry
SMOC	Standard Mean Ocean Chloride
SPLIce	Sample Processor for Life on Icy Worlds
SRM	Sulfate Reducing Microorganisms
SUDA	SUrface Dust Analyzer (Europa Clipper)
TSR	Thermochemical Sulfate Reduction
